# Mr. Smellie, on the Treatment of Ulcers at Sea

**Published:** 1808-10

**Authors:** Jacob Smellie

**Affiliations:** Assistant Surgeon. H. M. S. off Brest


### *To the Editors of the Medical and Physical Journal.*

GENTLEMEN,

IN a late Number of your Journal, my attention was naturally called to a communication by Mr. Warnock, " Assistant Surgeon of the Royal Navy," on the Treatment of Ulcers at Sea. He there states, that the practice of applying adhesive straps, is extended to all kind of ulcers, in all their stages, by the Practitioners in his Majesty's Navy. Now, as such a practice would carry ignorance and absurdity in its very front, I have thought it my duty to clear the practice of my brother officers from such an imputation.

To suppose that an *Assistant* Surgeon has drawn the statement, " that the unlimited use of adhesive straps in ulcers, is adopted throughout the generality of Men of War," from actual observation, would be to presume too much. That the practice he speaks of is not *universal,* I can state from my own experience; while I have very good authority for affirming, that it is not more generally or indiscriminately adopted in the Navy than on shore.

We have very great reason to distrust the sweeping assertion of Mr. W. " that" by far the majority of ulcers on board of a man of war *partake* of a scrophulous taint, or *are* attached to scorbutic patients, since we know that scurvy is now a very rare disease, especially on home stations.

That the compressing a deep ulcer, which discharges much matter, will occasion inflammation, and force the matter matter into the yielding cellular membrane, none will doubt; but many will certainly be surprised to learn, that Naval Surgeons are so blindly ignorant as to adopt such practice.

Had Mr. Warnock, (if he was determined to see his name in print) been content with publishing a single case, illustrative of the ill effects of this practice, *on board his own ship*, he might have had our praise, or at least, would have escaped our blame; but when we see one, the consideration of whose rank must convey to our minds the inexperience of youth, boldly accuse a most respectable body of gentlemen of depriving their country of the protection of its defenders, we can scarcely refrain our indignation at such unprincipled rashness.

I am, &c.

JACOB SMELLIE,

Assistant Surgeon.

*H. M. S. off Brest,*
*Aug.* 23, 1808.

